# Plastic Hatching Timing by Red-Eyed Treefrog Embryos Interacts with Larval Predator Identity and Sublethal Predation to Affect Prey Morphology but Not Performance

**DOI:** 10.1371/journal.pone.0100623

**Published:** 2014-06-26

**Authors:** Justin C. Touchon, Jeremy M. Wojdak

**Affiliations:** 1 Department of Biology, Boston University, Boston, Massachusetts, United States of America; 2 Smithsonian Tropical Research Institute, Balboa, República de Panamá; 3 Department of Biology, Radford University, Radford, Virginia, United States of America; Federal University of Rio de Janeiro, Brazil

## Abstract

Many animals respond to predation risk by altering their morphology, behavior, or life-history. We know a great deal about the cues prey respond to and the changes to prey that can be induced by predation risk, but less is known about how plastic responses to predators may be affected by separate plastic responses occurring earlier in life, particularly during the embryonic period. Embryos of a broad array of taxa can respond to egg- or larval-stage risks by altering hatching timing, which may alter the way organisms respond to future predators. Using the red-eyed treefrog (*Agalychnis callidryas*), a model for understanding the effects of plasticity across life-stages, we assessed how the combined effects of induced variation in the timing of embryo hatching and variation in the larval predator community impacted tadpole morphology, pigmentation and swimming performance. We found that *A. callidryas* tadpoles developed deeper tail muscles and fins and darker pigmentation in response to fish predators, either when alone or in diverse community with other predators. Tadpoles altered morphology much less so to dragonfly naiads or water bugs. Interestingly, morphological responses to predators were also affected by induced differences in hatching age, with early and late-hatched tadpoles exhibiting different allometric relationships between tail height and body length in different predator environments. Beyond induced morphological changes, fish predators often damaged tadpoles’ tails without killing them (i.e., sublethal predation), but these tadpoles swam equally quickly to those with fully intact tails. This was due to the fact that tadpoles with more damaged tails increased tail beats to achieve equal swimming speed. This study demonstrates that plastic phenotypic responses to predation risk can be influenced by a complex combination of responses to both the embryo and larval environments, but also that prey performance can be highly resilient to sublethal predation.

## Introduction

The ways that animals cope with predators have long been of interest to ecologists. A wide variety of organisms can respond to predators or herbivores by altering morphology, chemistry, coloration, behavior and life-history [1]–[4]. Such predator-induced phenotypic plasticity has most often been demonstrated in plants, aquatic invertebrates, and amphibian larvae [3], [4]. These organisms have provided ideal subjects for not only understanding the intricate ways that prey have evolved to cope with predation, but also to more accurately quantify and parameterize ecosystem functioning in light of plasticity. For example, plastic responses by prey to cues of predators can fundamentally alter ecosystem functioning and linkages across trophic levels, as well as buffer systems to perturbation [5].

Since most animals have complex life-cycles, the plastic responses that organisms exhibit in one life-stage may have carryover effects that can be seen during subsequent life-stages or that affect future plastic responses to risk or environmental heterogeneity. This has been best studied in the context of plastic larval responses affecting post-metamorphic morphology and behavior [6]. Organisms as diverse as bryozoans, gastropods, polychaetes, crustaceans, echinoderms, urochordates, insects and amphibians [7] can respond to larval conditions in ways that affect their post-metamorphic phenotype, for example in size, morphology, or larval period duration [3], [8]–[10]. Embryos can also exhibit plastic responses to risk and it is clear that those responses can carryover to effect the larval stage and beyond, although we are only now beginning to understand the nature of those lasting effects [9], [11]–[14].

In addition to indirect effects on inducing plastic responses in prey, predators primarily affect prey through direct consumption. However, an underappreciated but ecologically important effect of predators is sublethal predation. Predators often times only damage prey, which can have lasting impacts on morphology and life-history [15]–[17]. Perhaps the most well known examples of sublethal predation are lizards which autotomize their tails to escape predators [18], [19]. Although the lizard may survive the initial attack, the loss of the tail can decrease locomotor performance, reduce reproductive output and growth rate, and compromise foraging ability [19].

When predators co-occur, they can interact to have effects on prey that are different from what might be expected based on their individual effects [20], [21]. Depending on the specific members of a community and their particular habitat preferences or foraging styles, predators can enhance risk to prey (e.g., if predators co-occur and have similar foraging styles but do not interfere), reduce risk to prey (e.g., if predators interfere with or predate one another), or may have effects identical to what would be expected independently [22]. Thus, potential carryover effects of early life-stage plasticity may vary in different predator communities.

Anuran larvae are amongst the most common organisms in studies of predator-induced phenotypic plasticity. One of the most commonly reported responses of tadpoles to cues of predators is that they alter the shape and color of the tail [23]–[25]. These changes are generally thought to increase tadpole swimming performance and focus predator strikes to the tail, which is relatively expendable, at least in comparison to the body [26], [27]. Tadpoles can often live with only part of a tail, although swimming ability can be greatly reduced [17], [28]. This decrease in swimming ability is likely to affect escape success in subsequent interactions with predators [17]. However, induced morphological responses by tadpoles to their predators are not universal and tadpoles may exhibit fine-tuned predator-specific responses [3], [24], [29], [30].

**Figure 1:**
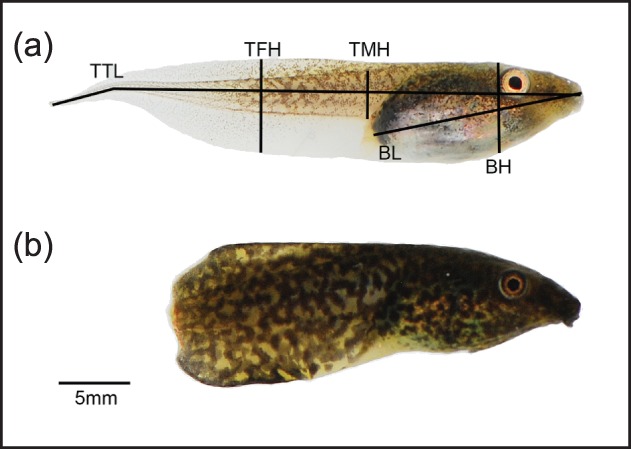
Morphological measurements taken on tadpoles and an example of sublethal predation. (a) A tadpole of our study species, the red-eyed treefrog *Agalychnis callidryas*, shown with the five lateral morphological variables measured. Variables were total tadpole length (total tadpole length), body length (body length), body height (body height), tail muscle height (tail muscle height), and tail fin height (TFD). (b) An *A. callidryas* tadpole reared with fish that has a maimed tail and increased pigmentation.

We investigated the effects of predator identity and sublethal predation on morphology and swimming performance using larvae of the red-eyed treefrog, *Agalychnis callidryas* (Cope; Fig. 1a). Red-eyed treefrog eggs respond to vibrations of predators (e.g., snakes or wasps) when being attacked and escape by hatching up to 30% early [31], [32]. Recent work by Touchon *et al.* [9] has demonstrated that this hatching plasticity has effects that last until metamorphosis. In addition, larval *A. callidryas* grow more slowly [33], reduce activity in response to cues from dragonfly naiad predators [11], and pre-metamorphic *A. callidryas* alter the timing of leaving the pond in response to cues from larval predators [34], [35]. However, it is currently unknown if there are lasting effects of hatching plasticity on the morphological development of prey in environments with different predator communities.

Our primary goal here is to test if induced variation in hatching timing of *A. callidryas* (i.e., plasticity during the egg-stage) affects later phenotypically plastic responses in morphology and pigmentation in response to free-roaming predators (i.e., plasticity during the larval stage). Our secondary goal is to determine if different predator species with different foraging modes, alone or in communities together, have similar effects on larval phenotypic plasticity. Our final goal is to test if variation in morphology resulting from the larval predator environment and sublethal predator attacks (tail maiming) translate into variation in swimming performance. We hypothesize that tadpoles will show morphological changes corresponding with predator lethality, that this will affect swimming performance, and that these morphological or behavioral effects may be affected by variation in hatching timing. Further, we hypothesize that tadpoles with damaged tails will have sub-optimal swimming performance compared to individuals with intact tails.

## Materials and Methods

As part of a separate experiment [36], *A. callidryas* tadpoles were raised in sixty 400-L cattle tanks in an open field facility of the Smithsonian Tropical Research Institute in Gamboa, Panama during August–October 2009. For full details see [36]. In short, *A. callidryas* tadpoles were raised in a full-factorial design of two hatching treatments and five predator treatments for a total of ten unique hatching–predator treatments fully replicated six times. Hatching age treatments were 4d post-oviposition or 6d post-oviposition (hereafter “early” or “late”), simulating the effects of snake predators [31]. Predator treatments were 1) no predator control, or three 2) fish [*Gambusia nicaraguensis* (Günther)], 3) dragonfly naiad [*Pantala flavescens* (Fabricius)], 4) small water bug [*Belostoma* sp.], or 5) all three predators in combination (one predator of each type for three total individuals). Hatching was induced by manually rubbing the egg clutch several times [31], [37], [38]. Mesocosms began with 50 tadpoles each and contained snails (*Physa* sp.), zooplankton, algae, and dead leaves from nearby trees. Tanks were set up in six spatial blocks. Because of limitations of available egg clutches, tanks were also established in three temporal blocks; two complete blocks were initiated on three separate dates (16 August, 3 September, and 23 September 2009). Each temporal block consisted of 7–13 clutches collected on each of two days (to obtain eggs of different ages that would hatch on the same day). To minimize possible genetic variation between clutches [11], all tadpoles hatched at a given time were hatched into a single container, mixed, and haphazardly sampled into groups of 50. The experiment was ended after 28 days and all tadpoles were removed with dipnets to quantify survival and morphology through the larval period. This research was conducted under Boston University IACUC protocol number 08-011 and research permits for work in Panama were granted by the Autoridad Nacional del Ambiente de Panamá (Permit SC/A-73-09).

Our three predators naturally co-occur at our research site and differ in functional morphology and foraging mode. *Pantala flavescens* is typically a sit-and-wait predator, with a benthic habitat domain, but will swim through the water column after prey [39]. Water bugs breathe air at the surface and generally ambush prey when they approach the surface. Mosquitofish are actively swimming, visual predators that readily prey upon amphibian eggs and larvae [40], [41], including *A. callidryas* tadpoles [31]. Since mosquitofish are not much larger than larval amphibians and become gape-limited, they often maim individual tadpoles but can kill them through multiple injuries (JMW & JCT, personal observations). Thus, these three predators represent a range of foraging modes and have distinct but partially overlapping habitat domains.

Upon removal from mesocosms, all tadpoles were photographed individually with a Nikon D70s digital camera with a Nikkor micro 105 mm lens (aperture: f13, shutter speed: 1/125 second). Tadpoles were placed in a narrow acrylic viewbox containing a small amount of water to allow the tadpole to float naturally and permit a clear lateral view of the animal. We took several photographs of each individual to ensure at least one where the full shape of the tadpole was visible. Photos included standard black and white squares for white balance, brightness, and contrast calibration, and a scale bar for size calibration. Electronic flashes were mounted in three positions to minimize shadows, and all camera settings were kept constant. The clear viewbox was kept in an opaque, darkened larger box to eliminate any ambient (and therefore potentially transient) light. We photographed a total of 1400 tadpoles.

For a subset of tadpoles (6–12 for each treatment combination), we also conducted swimming performance trials. Tadpoles were tested individually by placing them in a clear plastic box (33 cm×23 cm) containing aged tap water to a height of 2.5 cm, and a narrow swimming lane approximately 1.5 cm wide and 34 cm long (diagonal from corner to the other of the box). Swimming trials were filmed from below with a Sony Handycam HDR-SR7 digital video camera with lighting from above to create a clear silhouette of the tadpole. Tadpoles were placed at one end of the swimming lane and were prevented from swimming for ∼30 seconds by a barricade. The barricade was gently removed and the tadpole was stimulated to swim by touching it with a blunt probe at the back of tail. Each tadpole was probed up to six times (mean ± SE; 2.5±0.1 swims). We chose 1–5 swims where the tadpole clearly responded to the probe for analyses of swimming performance (1.89±0.1 swims). In total, we measured swimming performance in 91 tadpoles from the ten treatments (min. = 6, max. = 12). Analyses of swimming performance were conducted in ImageJ [42]. Videos were imported as a stack of images and the distance traveled from the start to the end of each swim was recorded as well as the time needed to travel that distance, which was used to calculate swimming speed (distance/time). In addition, we recorded the number of times the tadpole beat its tail during each swim, with each complete back and forth movement counted as two beats.

Lateral photographs were used for morphological analyses of tadpole shape. All measurements were obtained directly from digital photographs using ImageJ [42]. We measured Body Length (from the tip of the snout to the vent), Body Height (height of the body at the eyes), Tail Muscle Height (height of the tail muscle at the base of the body), Tail Fin Height (maximum height of the tail fin) and Total Tadpole Length (from the tip of the snout to the end of the tail). All measurements were log-transformed for subsequent analyses. Because the tail of some tadpoles had been maimed by predators (Fig. 1b), total tadpole length was not used in morphological analyses or swimming tests. Instead, we used relative tail length (tail length/body length) to test for swimming performance based on tail length while controlling for variation in body size.

Pigmentation of the body and tail was quantified by using the threshold function in ImageJ. Images were first standardized using the Colour Correct plug-in, in conjunction with white and black background colors in each image. We then tested multiple different thresholds until several naïve observers agreed that the selected “dark” pixels accurately matched “dark” areas of several example tadpole bodies. Pigmentation was quantified as the area (mm^2^) of dark pixels.

Tadpole survival varied substantially amongst treatments. This greatly affected the sample of tadpoles we were able to obtain for morphological analyses. In one treatment (Late hatching-Fish predator) there were only eight individuals (from three different blocks), whereas the other nine treatments had 155±18 (mean ±1 SD) tadpoles. Having a small sample makes it difficult to know if the values truly represent the mean values of the original distribution of tadpole sizes. To estimate the expected distribution of small samples of tadpoles from each treatment, we resampled 10,000 groups of eight tadpoles without replacement from each treatment and calculated the mean and 95% confidence interval for each treatment. Furthermore, since our predators were free-roaming and not caged, it can be difficult to discern between phenotype induction and selection. Thus, in the event of significantly different phenotypes we evaluated the distributions of tadpoles from different treatments in comparison to those from predator-free control tanks. If selection were the cause of apparent differences, we would expect the tadpoles with predators to represent a subset (i.e., one tail) of the overall distribution from the control distribution. If instead, phenotype induction was the primary cause of differences, we would expect tadpoles with predators to have a distribution of morphological characters outside the distribution of that of predator-free controls.

### Statistical analyses

All statistical analyses were conducted in R v2.15.0 [43]. Because we measured individuals that came from common environments (mesocosms) that were nested within blocks, we controlled for pseudoreplication by conducting linear mixed models which included block and tank nested within block as random effects, using the *lme4* package [44]. For analyses of tadpole morphology, these models were mixed analyses of covariance (ANCOVA) as recommended by [45] and [46]. body length was chosen as our covariate of tadpole “size” in all ANCOVAs, and we centered measurements prior to all analyses to reduce correlation between the estimated random slope and intercept [47]. Relative pigmentation was analyzed by the log-transformation of pigmentation area/total tadpole length. This allowed us to test for predator treatment and hatching age effects on the relative darkness of tadpoles given their overall size without needing to include a size covariate.

Because survival varied markedly across tanks, we tested for effects of hatching and predator treatments on body height, tail fin height and tail muscle height while controlling for variation in body length and the final number of tadpoles in each tank. Final tadpole density never had a significant effect and was therefore excluded from final models. Using only the tadpoles that had obviously damaged tails, we tested if relative tail length (controlling for body length) influenced tail muscle height or tail fin height. Lastly, to ensure that the tanks with low survival were not driving the results by themselves, in the event of three-way interactions we re-ran analyses while excluding the Fish Predator treatment. For analyses of swimming performance, we tested if tadpole speed or tail beats per cm swam were influenced by body length, and predator and hatching treatments. We also tested if speed or tail beats per cm swam were affected by relative tail length.

To estimate p-values and obtain the best model for each analysis, we began with the fully saturated model including all possible interactions and compared it with increasingly simplified nested models with likelihood ratio tests [48]. Post hoc comparisons among predator treatments were not feasible for analyses of morphology and swimming due to the inclusion of body length as a covariate. Post hoc comparisons of pigmentation were conducted using the *glht* function in the *multcomp* package [49].

## Results

### Tadpole morphology

**Figure 2:**
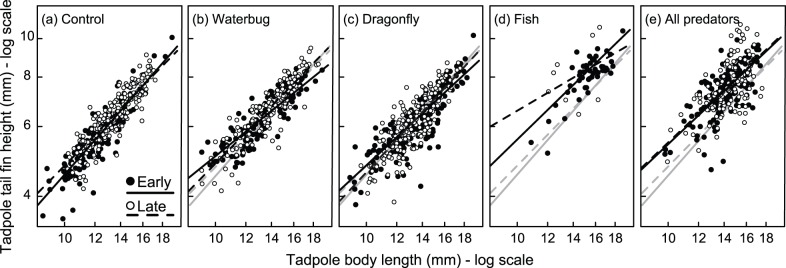
Hatching age and predator identity interact to affect tadpole tail fin height. The specific combination of hatching age (early or late, solid and dashed lines respectively) and predator treatment (panels a–e) determined the allometric scaling relationship between body length and tail fin height in *Agalychnis callidryas* tadpoles. Black lines are regression fits from a linear mixed model for each predator-hatching combination. Grey lines in panels (b)–(e) are the regression fits from the control treatments (panel a) and are shown to increase ease of comparison.

**Figure 3:**
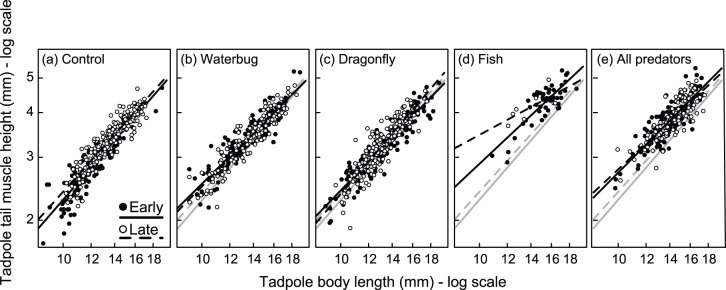
Predator identity affects tadpole tail muscle height. Predator treatments influenced the allometric scaling relationship between body length and tail muscle height in *Agalychnis callidryas* tadpoles. Data are also shown for each hatching age treatment (early or late) although this did not significantly affect tail muscle height. Black lines are regression fits from a linear mixed model for each predator-hatching combination. Grey lines in panels (b)–(e) are the regression fits from the control treatments (panel a) and are shown to increase ease of comparison.

*Agalychnis callidryas* tadpole morphology covaried with body size and was substantially affected by both predator and hatching treatments. Both tail fin height and tail muscle height increased with tadpole body length (Figs 2 and 3, Table 1). However, for tail fin height there was also a significant three-way interaction between body length, predator treatment and hatching treatment, such that the relationship between size and tail fin height was determined by the specific predator-hatching combination (Fig. 2, Table 1). Put another way, the effect of hatching age was dependent on the larval predator environment. In the absence of predators, there was a small difference in the allometry of early and late-hatched tadpoles such that smaller late-hatched tadpoles had larger tail fins. This effect was reversed when tadpoles were raised with dragonfly larvae or water bugs, greatly exacerbated with fish, and erased with all predators in combination (Fig. 2). Importantly, the three-way interaction between body length, predator treatment and hatching treatment on tail fin height was not driven by the low number of individuals in the fish treatment; removing the fish treatment from the analysis did not change the results in any appreciable manner (without the fish treatment: body length * Predator * Hatching, χ^2^ = 10.1, *P* = 0.018).

**Table 1:** Results of linear mixed models testing for effects of predator treatment (control, water bug, dragonfly, or fish alone or all three predators together) and hatching age (4 or 6 days post-oviposition), while controlling for effects of body length, on *Agalychnis callidryas* tadpole tail fin height and tail muscle height.

*Tail fin height*	Predictor	𝛘^2^	*P*-value
	Predators	45.6	<0.00001
	Hatching	1.8	0.18
	Body length	1346.8	<0.00001
	Pred * Hatch	0	1
	Pred * Body length	9.4	0.05
	Hatch * Body length	1.3	0.26
	Pred * Hatch * Body length	12.7	0.013
***Tail muscle height***	**Predictor**	**χ^2^**	***P*** **-value**
	Predators	55.4	<0.00001
	Hatching	2.6	0.11
	Body length	1721.2	<0.00001
	Pred * Hatch	5.8	0.21
	Pred * Body length	19.1	0.0008
	Hatch * Body length	0.11	0.75
	Pred * Hatch * Body length	8.0	0.092

Tadpole measurements used log-transformed data and body length measurements were centered prior to analyses.

There was no effect of hatching treatment on tail muscle height, but there were strong effects of predator treatment and the interaction between predator treatment and body length on tail muscle height (Fig. 3, Table 1). For a given body length, tadpoles raised with fish (either alone or in the all predator treatment) had deeper tail muscles than tadpoles from other treatments (Fig. 3). Similar to our results on tail fin height, removing the fish predator treatment did not alter the results whatsoever (without the fish treatment: body length * Predator, χ^2^ = 14.1, *P* = 0.003).

Our bootstrapped dataset of random samples of eight individuals from each treatment revealed that the differences in tail fin height and tail muscle height we detected in treatments with fish (Figs 2 and 3) were not due to a small sample size. We created 10,000 random datasets of eight individuals from each predator-hatching treatment combination, which revealed no overlap in the 95% confidence intervals of any other treatments with the actual mean values for tail muscle height or tail fin height in the Late-Fish treatment (Fig. S1). In addition, comparing the distributions of tadpole tail fin height and tail muscle height from the two treatments with fish with the other three treatments clearly demonstrates that tadpoles reared in environments with fish did not simply represent a subset of overall tadpoles that were selected for via predation, but represent individuals with an induced phenotype (Figs S2 and S3).

Body height was strongly and positively associated with body length, and there was a weak effect of predators inducing relatively shallower bodies than controls (body length, χ^2^ = 1157.3, *P*<0.00001, Predator, χ^2^ = 9.3, *P* = 0.054). There was no effect of hatching treatment on body height (χ^2^ = 0.3, *P* = 0.6) nor did hatching age interact with any other variables (all interactions, *P*>0.09).

**Figure 4:**
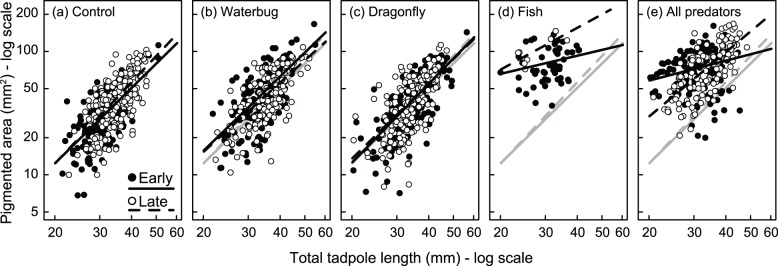
Tadpoles had darker pigmentation if raised with fish. Relative *Agalychnis callidryas* body pigmentation for tadpoles raised in predator-free control tanks or in one of four predator treatments (dragonfly naiads, water bugs or fish alone or all three predators together). Tadpoles were significantly more pigmented in the two treatments containing fish. Relative pigmentation was defined as log (Area of pigmentation/total tadpole length).

The relative proportion of a tadpoles body that was darkly pigmented was significantly affected by predator treatment but not by hatching age or the interaction between hatching age and predator treatment (Fig. 4; Predator, χ^2^ = 55.5, *P*<0.00001, Hatching: χ^2^<0.01, *P* = 0.99; Predator * Hatching, χ^2^ = 3.7, *P* = 0.45). Pigmentation was greater in tadpoles from fish or multiple predator tanks, whereas tadpoles raised with dragonfly naiads or water bugs were not different from controls (all post hoc comparisons between fish or all predator treatments and other treatments, *P*≤0.003, all other post hoc comparisons *P*≥0.23).

### Tadpole swimming performance

**Figure 5:**
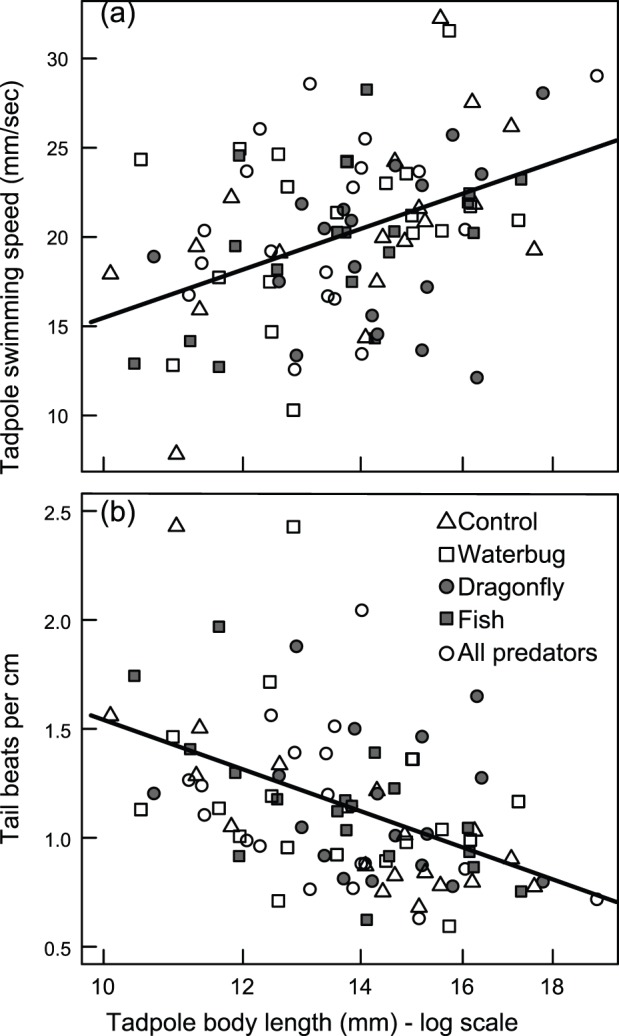
Tadpole swimming performance was affected by body size. *Agalychnis callidryas* tadpole (a) swimming speed increased with body length, whereas (b) the number of beats per centimeter of distance swam decreased with body length. Neither aspect of swimming was affected by predator treatments.

All tadpoles from treatments with fish that we tested for swimming performance had at least part of their tail missing. No tadpoles in the control, water bug or dragonfly treatments were missing any of their tail. Despite variation in the amount of tail that tadpoles had, swimming speed and number of tail beats per cm increased with body length but neither was affected by predator or hatching treatments or their interaction (Fig. 5; speed: body length, χ^2^ = 14.2, *P* = 0.0002, Predator, χ^2^ = 1.5, *P* = 0.8, Hatching, χ^2^ = 0.003, *P* = 0.96, all interactions, *P*>0.2; beats per cm: body length, χ^2^ = 17.3, *P<*0.0001, Predator, χ^2^ = 0.1, *P* = 0.99, Hatching, χ^2^ = 0.6, *P* = 0.46, all interactions, *P*>0.13). Thus, tadpoles with bigger bodies (excluding the tail) swam faster than smaller tadpoles, but tadpoles that had maimed tails did not swim significantly slower than tadpoles from treatments without fish. Controlling for body length, swimming speed was not affected by tail muscle height, tail fin height, or body height (tail muscle height: χ^2^ = 1.1, *P* = 0.3, tail fin height: χ^2^ = 1.9, *P* = 0.2, body height: χ^2^ = 0.9, *P* = 0.3). Swimming effort, i.e. tail beats per cm swam, decreased with increasing tail muscle height but was not affected by tail fin height (tail muscle height: χ^2^ = 5.2, *P* = 0.02, tail fin height: χ^2^ = 1.5 = 0.2, body height: χ^2^ = 0.2, *P* = 0.6).

**Figure 6:**
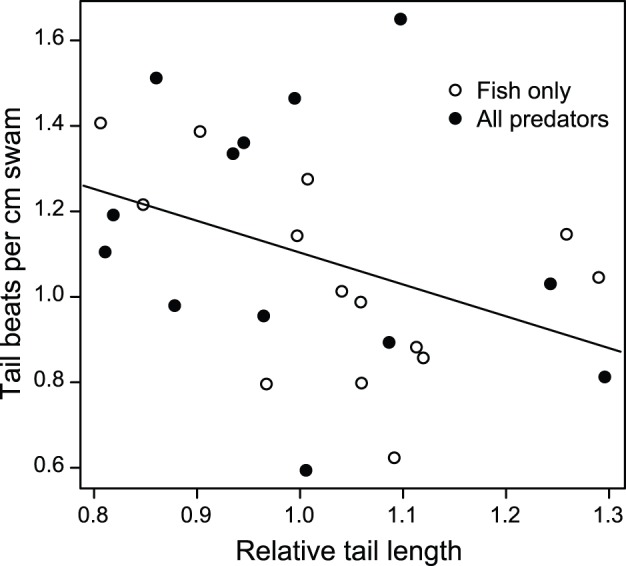
Tadpoles with shortened tails swam as fast as tadpoles with longer tails. *Agalychnis callidryas* tadpoles that were reared with fish, either in the fish only predator treatment or the all predator treatment, had substantially shortened tails in comparison to tadpoles from other predator treatments. Tadpoles with shorter tails relative to their body size also exerted more tail beats per centimeter of distance swam.

Among only the tadpoles with maimed tails, tadpoles with relatively smaller tails (i.e., tails that were more damaged) did not swim significantly slower than tadpoles with relatively longer tails (χ^2^ = 2.1, *P* = 0.15). The relative length of the tail did however affect swimming effort; tadpoles with relatively shorter tails swam using more beats per cm of distance traveled than tadpoles with relatively longer tails (Fig. 6; χ^2^ = 4.7, *P* = 0.03).

## Discussion

There were two main results from our study. First, *A. callidryas* larvae altered the size of their tail fins in response to the larval predator community, but the specific nature of this morphological response was influenced by when they had hatched as embryos. Tadpoles also altered tail muscle height in response to predators, although this was not affected by variation in hatching timing. Secondly, although fish reduced the size of *A. callidryas* tadpoles’ tails dramatically, we found that this had no effect on swimming speed because tadpoles increased their effort proportionally, allowing them to maintain typical locomotor performance.

The sort of plastic responses to environmental variation we document here are being increasingly found across the Bilateria [50] and are likely pervasive in nature. Furthermore, these responses early in the life of an animal can have unexpected lasting effects. For example, Touchon *et al.* [9] recently found that a two-day (30%) change in hatching timing of *A. callidryas* affected larval viability and metamorphic phenotype. We had no specific *a priori* predictions about the effects of hatching plasticity here because it is not currently well understood why such lasting effects are present. In anuran larvae, different types of stressful conditions (e.g., predators, pond drying, etc) can have differing effects on tadpole growth and development [6]. One might have expected that early hatched individuals would develop stronger morphological or pigmentation responses to predators because they are exposed to cues at an earlier point in development, at which point they are more vulnerable to most aquatic predators [51]. Instead, we found that variation in tail fin height was dependent on the specific combination of hatching age and predators in the environment (Fig. 2). In essence, early hatched tadpoles had shallower tail fins in the absence of predators but the allometry between tail fin size and body size changed with different predators. With water bugs and dragonfly larvae, tadpole tail fin height increased more rapidly with increasing body length for late-hatched tadpoles (i.e., the slope of the regression between body length and tail fin height was steeper). However, the opposite pattern was true when tadpoles were reared with fish. Most interestingly, there were no morphological differences between early and late-hatched tadpoles in the presence of the diverse predator community, the most realistic scenario. This result may indicate that subtle effects of hatching age are obscured in complex environmental conditions and further emphasizes the context dependency of carryover effects of early life-stage plasticity.

Fish predators had the strongest lethal effects in our experiment, reducing density by 97% in one treatment [36]. Thus, it might be easy to dismiss the results we found as aberrant and due to sample size issues. However, even after removing the fish predator treatment entirely, we still found an interaction between tadpole allometry, hatching age and predator treatment, clearly indicating that the one treatment with few individuals was not driving the patterns we found. Indeed, treatments lacking fish would never be expected to produce individuals with tail muscles or fins as large as the ones from our fish treatments (Fig. S1). Furthermore, the tadpoles from tanks with fish do not merely represent a subset of tadpoles that were left after selection by predators but instead clearly represent individuals with a different phenotype (Figs S2 and S3). With our experimental design, we cannot however distinguish between phenotype induction due to predators in the environment [23]–[25] versus induction resulting sublethal predation or other physical interactions with the predators themselves.

In the multiple predator treatment, predation on tadpoles was reduced, potentially as a result of interference competition or intimidation effects between water bugs and fish [36]. In accordance with this variation in risk, tadpole morphological responses were strongest with fish alone, followed by the all-predator treatment. However, it was clearly not only predation risk that tadpoles responded to, but was instead predator identity. Tadpole survival was similar in the multiple predator treatment and the water bug only treatment [36], but tadpoles only developed enlarged tail muscles and darkened pigmentation in the treatment containing fish, indicating that it was not risk alone that tadpoles responded to (Fig. 3). Even though risk was reduced with multiple predators, tadpoles responded to the most dangerous predator in the community [21].

Although the majority of studies have found that tadpoles increase tail height in response to predators [3], this is not universal. For example, in one study with multiple tadpole species, *Anax* dragonfly naiads and *Belostoma* water bugs caused tadpoles to develop relatively deeper or shallower tail fins, or had no effect, depending on the specific anuran species being studied [23]. It might naively seem that being able to swim faster would be advantageous when facing any predator; however, the advantage of speed when facing different predator foraging modes (sit-and-wait, active pursuit, etc.) might not be equivalent, and there are tradeoffs associated with different defensive morphologies. In general, the larger tail fins that aid in turning and initial burst speed–most useful for dodging an attack from a sit-and-wait predator but less useful for predators that pursue prey–can decrease overall swimming speed, and thus prey species do not respond to all predators in the same manner [52]–[55].

Of the three predators used in our study, fish (*G. nicaraguensis*) are the only ones that are likely to actively pursue tadpoles for an extended period of time or distance. *Pantala flavescens*, our dragonfly naiad predator, is an active predator that inhabits the bottom of ponds [56] and will swim through the water column to attack tadpoles [39]. However, *P. flavescens* do not chase tadpoles for extended periods and they become gape limited as *A. callidryas* grow [33]. The small species of water bugs we used similarly has difficulty capturing large *A. callidryas* tadpoles [36]. Thus it may be that *A. callidryas* does not respond morphologically to predators that are only a threat during the first part of the larval period. Lastly, we cannot rule out that the enlarged tail muscles and fins in treatments with fish were a direct result of being actively chased in the mesocosm environment.

Many of the *A. callidryas* tadpoles that were not consumed by fish suffered sublethal predation, with a substantial portion of their tails being consumed without actually killing the tadpole. Sublethal predation is common in nature and has been linked to the presence of amphibian deformities [15], decreased tadpole survivorship [17], [57], and decreased sustained swimming performance in fish [17], [52]. Surprisingly, we found that variation in tadpole tail length as a result of sublethal predation did not affect swimming speed [58]. Two previous studies that surgically shortened the tail if tadpoles found that substantially reducing the length of the tail decreased swimming speed [17], [28]. In contrast to their results, we found that tadpoles that had been attacked but not killed by fish did not swim slower but did increase swimming effort (Fig. 6). In addition, we found that tadpoles with smaller tail muscles also gave a stronger swimming effort; they utilized more beats per cm than tadpoles with larger (and thus presumably stronger) tail muscles. Unlike Figiel Jr. and Semlitch [17] and Van Buskirk and McCollum [28], we do not have an objective measure of how much the tail was shortened by nonlethal fish predation, since we do not know the length of the tail before injury nor when injuries occurred during the experiment. Furthermore, it is not unreasonable to think that there may be differences in swimming performance between animals which have been injured and have healed over days or weeks, as opposed to animals surgically manipulated and tested immediately [17] or after recovering from anesthesia [28].

Why did tadpoles with damaged tails swim harder than expected? One possible explanation is purely mechanistic. As tadpoles grow and the tail becomes larger, it presumably becomes more difficult to move. Thus, more tail musculature is needed for swimming. When the tail subsequently becomes shortened by a predator, the injured tadpole is likely to have disproportionately more musculature than is needed to power a small tail, thereby permitting it to beat its tail harder than expected and maintain high levels of swimming speed. Alternatively, tadpoles with damaged tails may have a lower threshold for responding to risk, and may respond with greater exertion, because they have already experienced predation risk. The initial predation attempts by fish may shift the threshold needed to elicit a flight response. Such learning has been demonstrated in fishes and amphibians, but generally based on chemical cues and not resulting from direct experience [59]–[61]. These two hypotheses are not mutually exclusive and future work should attempt to tease these apart.

### Summary

Plasticity in early life history, specifically in the timing of hatching, is widespread and is increasingly being found across a wide array of taxa [50]. We demonstrate not only that *A. callidryas* tadpoles demonstrate predator-specific changes in the size of tail fins and muscles, but also that those predator-induced responses can be altered by a two day change in hatching timing. Furthermore, variation in the composition of the predator community and therefore the risk posed to prey, alters prey phenotypes in direct and indirect ways, by physically maiming prey individuals and inducing phenotypic changes. Quantifying the effects of such plasticity is key to understanding the potential role that flexible phenotypes may play in modifying communities.

## Supporting Information

Figure S1 **Means ±95% confidence intervals for (a) tail fin height and (b) tail muscle height for 10,000 randomly sampled groups of eight** ***Agalychnis callidryas*** **tadpoles from each hatching age/predator treatment combination.** Black bars indicate the late-hatched/fish only treatment that had 8 surviving individuals at the end of the experiment. Red bars indicate other treatment combinations that had fish.
(PDF)

Figure S2 **The distribution of tail fin heights of early hatched – control tadpoles as compared to early hatched tadpoles raised with a) fish, b) all predators, c) dragonflies or d) water bugs.** Tadpoles raised with fish, either alone or in the all predator treatment, had larger tail fins that did not merely represent a subset of the overall distribution of tadpoles expected in the controls. Tadpoles raised with dragonflies or water bugs had tail fins very similar to controls.
(PDF)

Figure S3 **The distribution of tail muscle heights of early hatched – control tadpoles as compared to early hatched tadpoles raised with a) fish, b) all predators, c) dragonflies or d) water bugs.** Tadpoles raised with fish, either alone or in the all predator treatment, had larger tail muscles that did not merely represent a subset of the overall distribution of tadpoles expected in the controls. Tadpoles raised with dragonflies or water bugs had tail muscles very similar to controls.
(PDF)
