# Supplementary figures and images for: Plastic Hatching Timing by Red-Eyed Treefrog Embryos Interacts with Larval Predator Identity and Sublethal Predation to Affect Prey Morphology but Not Performance
Source: PLoS One. 2014 Jun 26;9(6):e100623. doi: 10.1371/journal.pone.0100623 (PMC4072661; doi:10.1371/journal.pone.0100623)

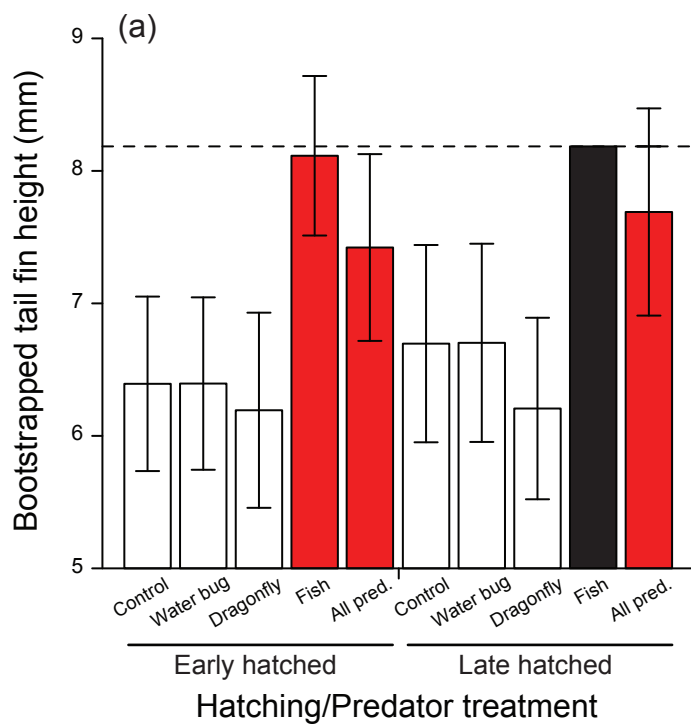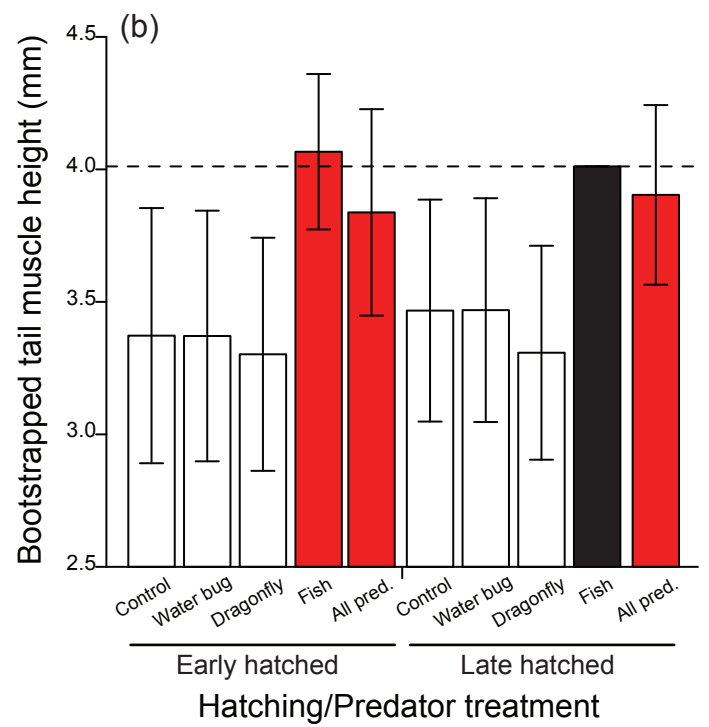

Supporting Figure S1

Supplement: Figure S1 — Means ±95% confidence intervals for (a) tail fin height and (b) tail muscle height for 10,000 randomly sampled groups of eight Agalychnis callidryas tadpoles from each hatching age/predator treatment combination. Black bars indicate the late-hatched/fish only treatment that had 8 surviving individuals at the end of the experiment. Red bars indicate other treatment combinations that had fish. (PDF) [file pone.0100623.s001.pdf]

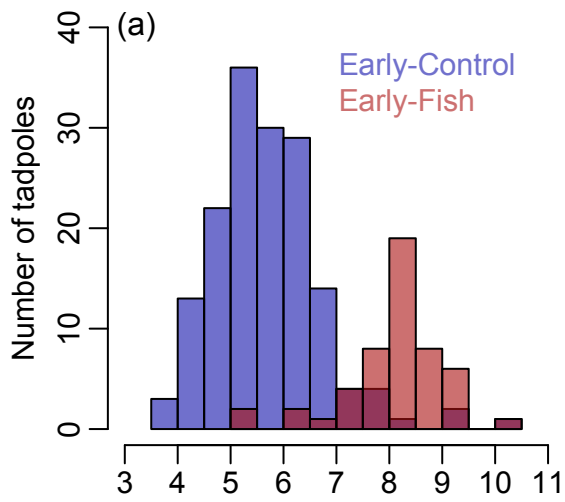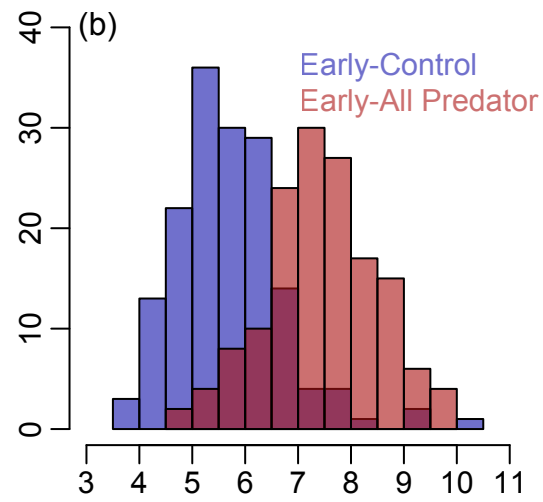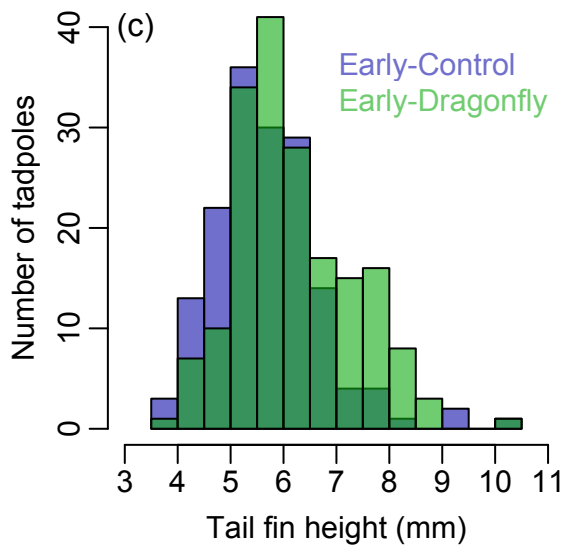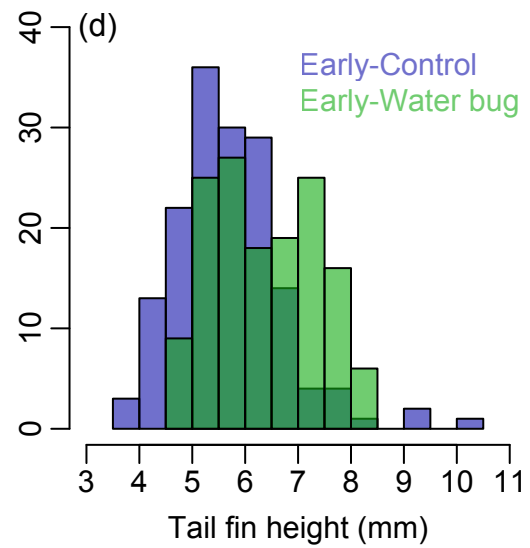

Supplement: Figure S2 — The distribution of tail fin heights of early hatched – control tadpoles as compared to early hatched tadpoles raised with a) fish, b) all predators, c) dragonflies or d) water bugs. Tadpoles raised with fish, either alone or in the all predator treatment, had larger tail fins that did not merely represent a subset of the overall distribution of tadpoles expected in the controls. Tadpoles raised with dragonflies or water bugs had tail fins very similar to controls. (PDF) [file pone.0100623.s002.pdf]

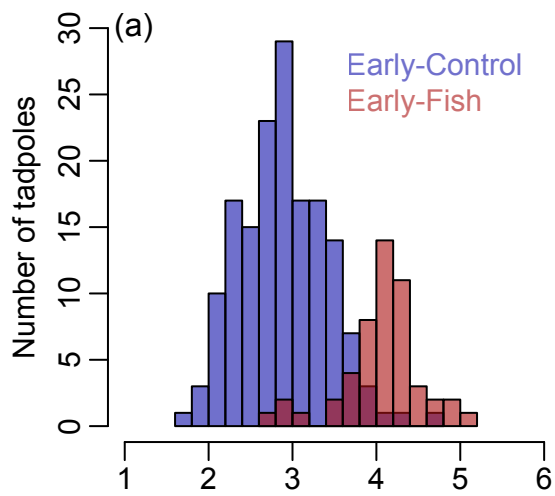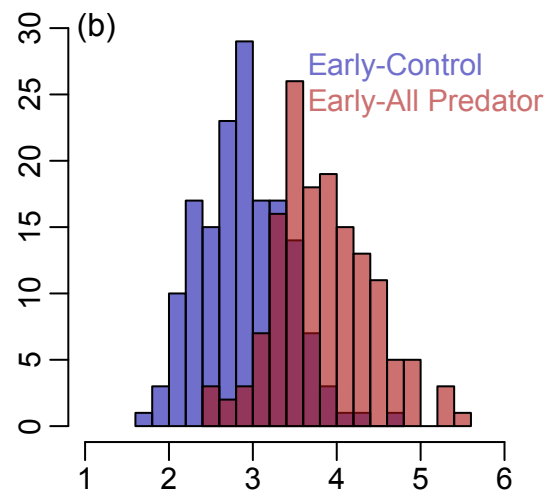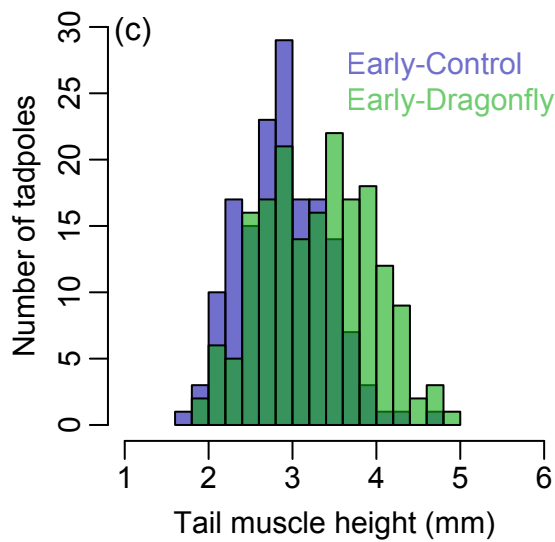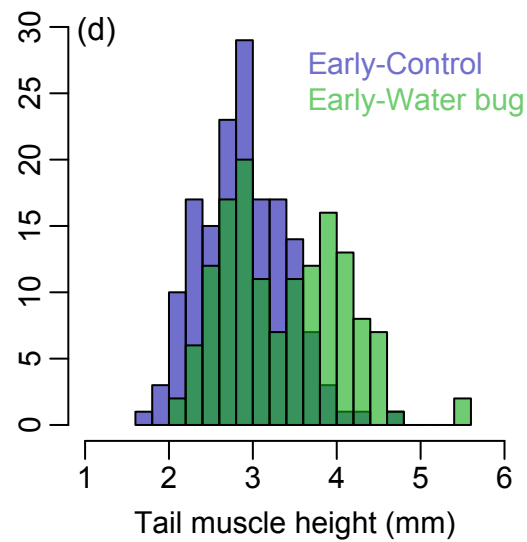

Supplement: Figure S3 — The distribution of tail muscle heights of early hatched – control tadpoles as compared to early hatched tadpoles raised with a) fish, b) all predators, c) dragonflies or d) water bugs. Tadpoles raised with fish, either alone or in the all predator treatment, had larger tail muscles that did not merely represent a subset of the overall distribution of tadpoles expected in the controls. Tadpoles raised with dragonflies or water bugs had tail muscles very similar to controls. (PDF) [file pone.0100623.s003.pdf]
